# Increased Microbial Diversity and Decreased Prevalence of Common Pathogens in the Gut Microbiomes of Wild Turkeys Compared to Domestic Turkeys

**DOI:** 10.1128/aem.01423-21

**Published:** 2022-03-08

**Authors:** Julia Craft, Hyrum Eddington, Nicholas D. Christman, Weston Pryor, John M. Chaston, David L. Erickson, Eric Wilson

**Affiliations:** a Department of Microbiology and Molecular Biology, Brigham Young University, Provo, Utah, USA; b Department of Plant and Wildlife Sciences, Brigham Young University, Provo, Utah, USA; Universidad de los Andes

**Keywords:** *Escherichia coli*, microbiome, poultry production, wild turkey, poultry pathogens

## Abstract

Turkeys (Meleagris gallopavo) provide a globally important source of protein and constitute the second most important source of poultry meat in the world. Bacterial diseases are common in commercial poultry production, causing significant production losses for farmers. Due to the increasingly recognized problems associated with large-scale/indiscriminate antibiotic use in agricultural settings, poultry producers need alternative methods to control common bacterial pathogens. In this study, we compared the cecal microbiota of wild and domestic turkeys, hypothesizing that environmental pressures faced by wild birds may select for a disease-resistant microbial community. Sequence analyses of 16S rRNA genes amplified from cecal samples indicate that free-roaming wild turkeys carry a rich and variable microbiota compared to domestic turkeys raised on large-scale poultry farms. Wild turkeys also had very low levels of Staphylococcus, Salmonella, and Escherichia coli compared to domestic turkeys. E. coli strains isolated from wild and domestic turkey cecal samples also belong to distinct phylogenetic backgrounds and differ in their propensity to carry virulence genes. E. coli strains isolated from factory-raised turkeys were far more likely to carry genes for capsule (*kpsII* and *kpsIII*) or siderophore (*iroN* and *fyuA*) synthesis than were those isolated from wild turkeys. These results suggest that the microbiota of wild turkeys may provide colonization resistance against common poultry pathogens.

**IMPORTANCE** Due to the increasingly recognized problems associated with antibiotic use in agricultural settings, poultry producers need alternative methods to control common bacterial pathogens. In this study, we compare the microbiota of wild and domestic turkeys. The results suggest that free-ranging wild turkeys carry a distinct microbiome compared to farm-raised turkeys. The microbiome of wild birds contains very low levels of poultry pathogens compared to that of farm-raised birds. The microbiomes of wild turkeys may be used to guide the development of new ways to control disease in large-scale poultry production.

## INTRODUCTION

Turkeys (Meleagris gallopavo) evolved approximately 11 million years ago and are one of the first birds domesticated in the Americas (1–3). Although domesticated thousands of years ago, turkeys have remained generally very similar to their wild relatives until relatively recently (4, 5). In the past ∼70 years, intensive selective breeding of turkeys has resulted in dramatic changes in commercially raised birds compared to their wild relatives, leading to a genome that is much less diverse than those of many other agricultural species (4). These genetic changes as well as advancements in production practices have resulted in domestic birds maturing much more quickly and reaching three times the body mass of wild birds at maturity (6). Domestic turkeys are now the second most important source of poultry in the world, with the United States producing ∼250,000,000 turkeys and ∼7,000,000,000 pounds of turkey meat in 2019 (7).

Relatively few studies have been published comparing the microbiomes of wild animals and their domesticated kin. However, the limited literature on this topic has overwhelmingly shown that the microbiomes of captive and wild animals vary dramatically (8–15). The observed differences in microbial communities between wild and captive animals have led to calls for more research on the microbiomes of additional wild animals (16, 17).

The gut microbiome of poultry is known to contribute to efficient growth as well as bird health (11, 18–21). The microbiome of commercially raised poultry is undoubtedly influenced by production practices such as crowded conditions, diet, and antibiotic use. Several studies have characterized the gut microbiomes of domestic turkeys in a variety of experimental and agricultural settings (20, 22–25); however, very few studies have focused on the microbiomes of wild turkeys (11).

In an effort to better characterize the potential effects of the gut microbiota on turkey health and disease, we compared the cecal microbiota from factory-raised domestic, free-ranging domestic, and free-ranging wild turkeys. Sequencing of the V4 region of the 16S rRNA gene was used to determine the abundance of multiple taxa in the ceca of individual birds within each group. Additional experiments were designed to determine the prevalence of bacterial taxa that are common pathogens of commercially raised turkeys. These studies indicate that beta diversity values within the microbiota are significantly different among factory-raised domestic turkeys, free-ranging domestic turkeys, and free-ranging wild turkeys. Several common pathogens associated with commercial poultry production (Escherichia coli, Salmonella species, and Staphylococcus species) were infrequent or absent in the cecal microbiota of free-ranging wild turkeys. E. coli strains found in wild turkeys were found to be genetically diverse and carry fewer virulence-associated genes than strains found in factory-raised birds.

## RESULTS

**FIG 1:**
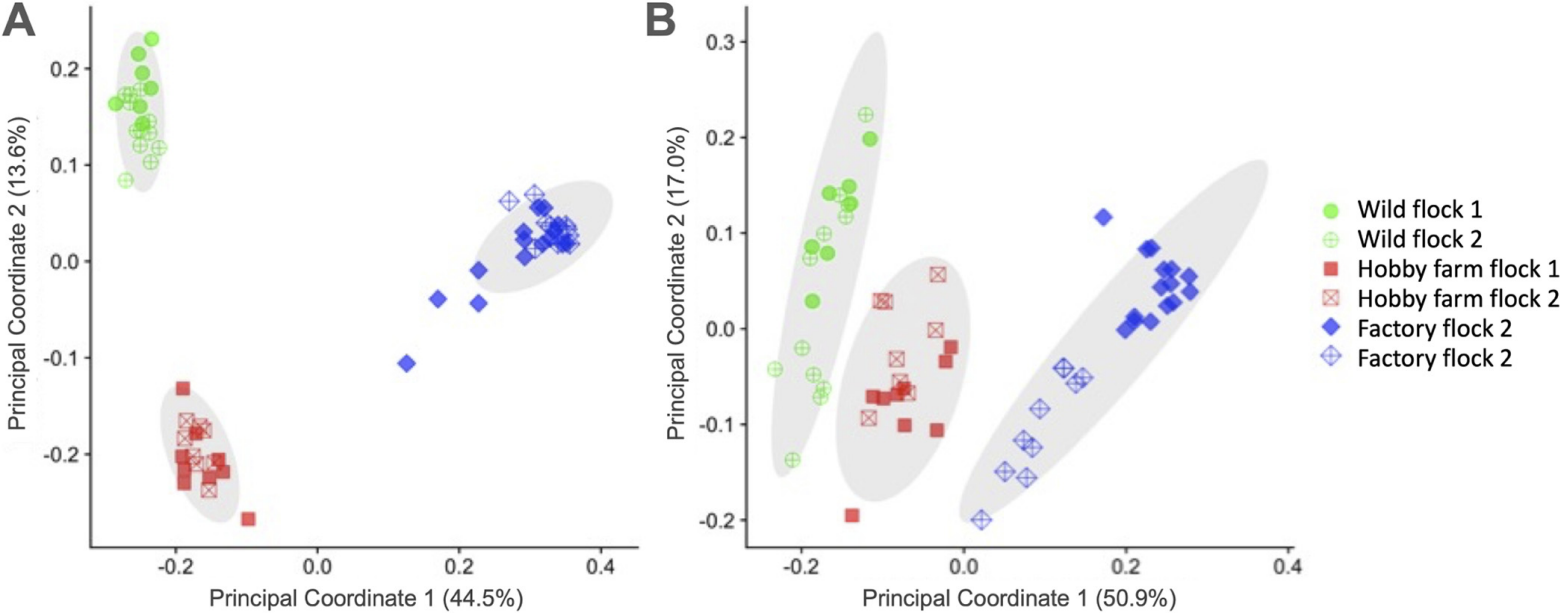
Principal-component analysis demonstrates that the cecal microbiota of turkeys cluster according to bird provenance. Weighted (A) and unweighted (B) UniFrac distance plots of the different flocks are colored according to the animals’ provenance.

To explore potential differences in the microbiota of wild versus domestic turkeys, a 16S rRNA gene survey of the cecal microbiota was performed. A total of 4,070,891 bacterial reads were obtained, with an average of 53,564 reads per sample and 3,069 amplicon sequence variants (ASVs). We performed principal-coordinate analysis (PCoA) and permutational multivariate analysis of variance (PERMANOVA) of weighted UniFrac distances to compare the microbiota compositions of different flocks of turkeys. At a 13,000-read subsampling depth, PERMANOVA of UniFrac distances revealed significant differences in the microbiota of the samples within provenance and flock (Table 1). The clustering of samples on PCoA ordinations visually depicted these statistical differences, where principal coordinates 1 and 2 separated the samples into three general groups that matched the provenance of the samples when analyzed by both weighted and unweighted UniFrac distances (Fig. 1). Follow-up weighted and unweighted UniFrac analyses confirmed that there were flock-specific effects when each provenance was analyzed separately, except for birds from factory-raised flocks, analyzed by weighted UniFrac (Table 1). The finding that all flocks differed in beta diversity, except those raised in commercial production facilities, is likely a reflection of the highly standardized nature of commercial poultry production.

**TABLE 1:**
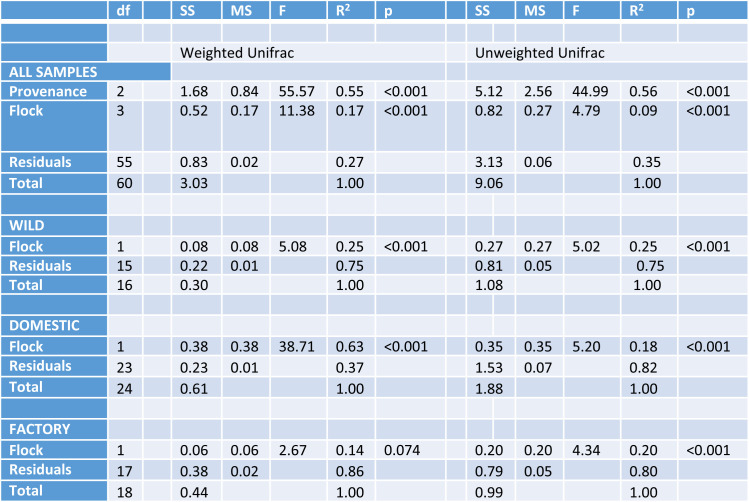
PERMANOVA for different groups of samples^*a*^

a df, degree of freedom; SS, sum of squares; MS, mean of squares.

**FIG 2:**
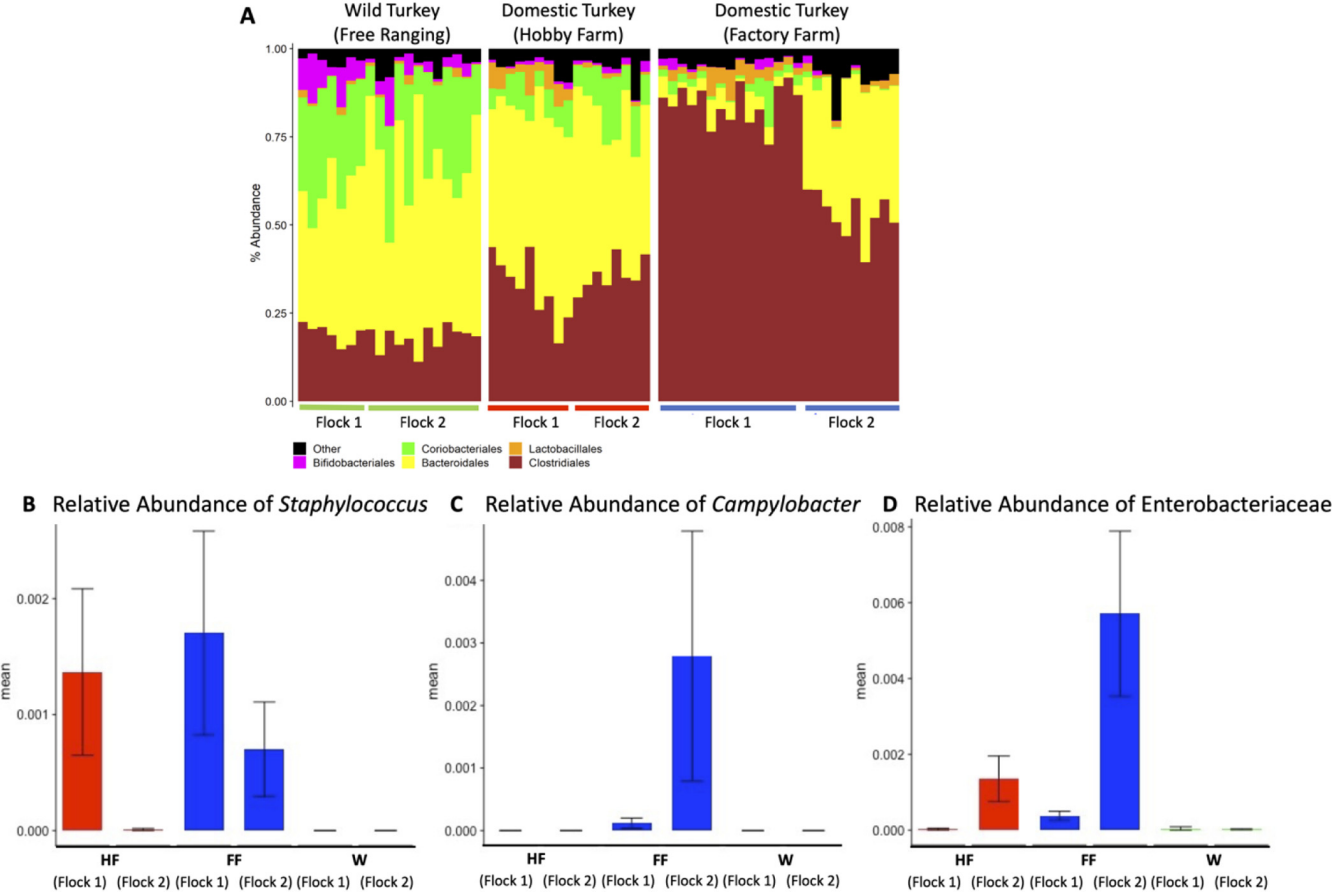
(A) Taxon plot of flocks, grouped by provenance. Order-level assignments above a 2% total relative abundance are shown individually. (B to D) Relative abundances of groups of ASVs (B and C) or an individual ASV (D) from the 16S sequencing data set. Samples are grouped according to flock and colored by provenance. Red, hobby farm (HF); blue, factory farm (FF); green, wild bird (W). (B and C) Sum of reads from multiple ASVs that were each assigned to the Staphylococcus or Campylobacter genus. (D) Reads from a single ASV that could not be assigned below the *Enterobacteriaceae* family.

We also evaluated the variation in the microbiota compositions of the different flocks. At the order level, there were significant differences in the numerical density of the most abundant bacterial taxa (Fig. 2A). For example, in the factory-raised birds, *Clostridiales* were the most abundant taxon (71.7% ± 3.3%), much more abundant than in free-ranging domestic turkeys raised on hobby farms (33.8% ± 1.8%) or wild turkeys (18.3% ± 0.7%). The lower *Clostridiales* read counts in the free-ranging domestic and wild turkey flocks were largely offset by relative increases in *Bacteroidales* and *Coriobacteriales*. The abundances of these reads were all significantly different between provenances by analysis of composition of microbiomes (ANCOM) (see Table S2 in the supplemental material). However, despite these differences in the abundances of different taxa, the variation in alpha diversity between flocks was not related to the flocks’ provenance (Table 2). Therefore, key differences in numerical composition at high taxonomic levels did not necessarily reflect low-level differences in diversity.

**TABLE 2:**
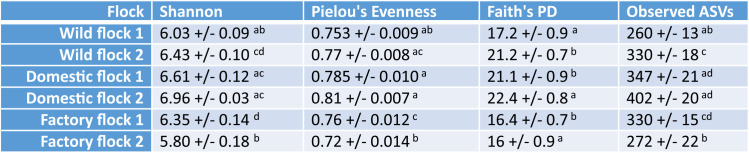
Alpha diversity metrics on a per-flock basis^*a*^

a Data are shown as means +/− standard errors of the means (SEM). Different letters next to the SEM represent significant differences between flocks for each metric and were determined by a Kruskal-Wallis test. PD, phylogenetic diversity.

To better understand the potential relationship between flock provenance and the carriage of potential pathogens, we next focused on the relative abundances of taxa of veterinary and medical importance by identifying ASVs that best matched known bird pathogens. V4 sequences representing Staphylococcus sp. were most prevalent in samples from commercially raised birds. Staphylococcus DNA was also detected in one flock of domestic free-ranging turkeys. Detectable levels of Staphylococcus DNA were not found in any samples from wild birds. Similarly, Campylobacter DNA was identified only in factory farm-raised birds. The abundance of Campylobacter DNA in some birds was suggestive of heavy colonization; however, it was undetected in other birds within the same flock (Fig. 2B and C).

One limitation of our approach is that without whole-genome data, the short region that we sequenced cannot distinguish known pathogens from similar bacteria with identical sequences across the 16S V4 region. Measurable levels of the family *Enterobacteriaceae* were abundant in samples from both factory-raised flocks and one free-ranging domestic flock (Fig. 2D). The *Enterobacteriaceae* are a large family of bacteria that include Escherichia coli as well as other pathogens, including Salmonella. The V4 region of the 16S rRNA gene does not resolve E. coli or Salmonella from other *Enterobacteriaceae*, which prevented us from estimating E. coli or Salmonella abundance in these animals through V4 sequencing alone. As E. coli and Salmonella are common pathogens in domestic poultry production, we further investigated the prevalence of these potential pathogens in wild and factory-raised turkeys.

**FIG 3:**
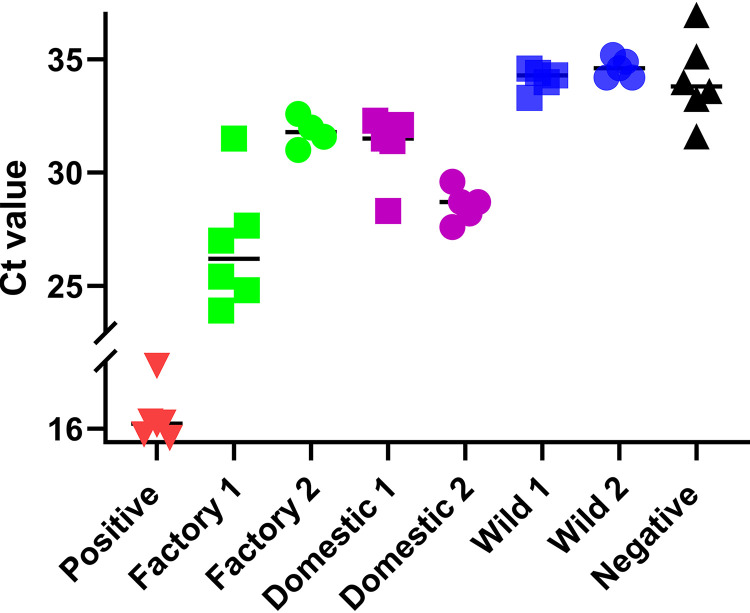
Cecal contents of factory-raised and free-ranging domestic turkeys contain high levels of E. coli DNA compared to those of wild birds. Quantitative PCR was performed for the *ybbW* gene (E. coli specific) detected in total genomic DNA isolated from the ceca of factory-raised domestic, free-range domestic, and wild turkeys. Threshold cycle (*C_T_*) values from individual birds are shown on the *y* axis. Average *C_T_* values of each group are indicated by a horizontal bar. *C_T_* values of the positive control (100% E. coli genomic DNA) and negative control are indicated on the *x* axis.

The presence of Salmonella DNA was detected by PCR targeting the Salmonella-specific gene *invA* in total DNA isolated from cecal samples of individual birds. Of 14 samples tested from factory-raised birds, 11 tested positive for *invA*. Conversely, none of the 11 samples collected from wild birds tested positive for the presence of the *invA* gene, suggesting that factory-raised turkeys more frequently contain Salmonella in their digestive tracts than wild turkeys. To determine the relative abundance of E. coli in cecal samples, a quantitative PCR (qPCR) assay targeting the E. coli-specific gene *ybbW* (26) was used. Genomic E. coli DNA was clearly present in the total DNA samples obtained from commercially raised turkeys and hobby farm-raised turkeys. Conversely, E. coli DNA in samples from wild turkeys was below the limit of detection of the assay (Fig. 3). We also plated cecal samples on MacConkey agar to enrich for the growth of enteric bacteria. Although not detectable by qPCR, we were able to isolate colonies characteristic of E. coli from wild turkey cecal samples. Their identity as E. coli was subsequently verified by amplification of the *ybbW* gene. E. coli was readily cultured from the ceca of factory-raised turkeys. In addition to colony growth consistent with E. coli (pink colonies), white colonies were also observed growing on MacConkey agar. These white colonies were not studied further or collected; however, based on growth on MacConkey agar, these colonies were likely Salmonella or other enteric bacteria.

**FIG 4:**
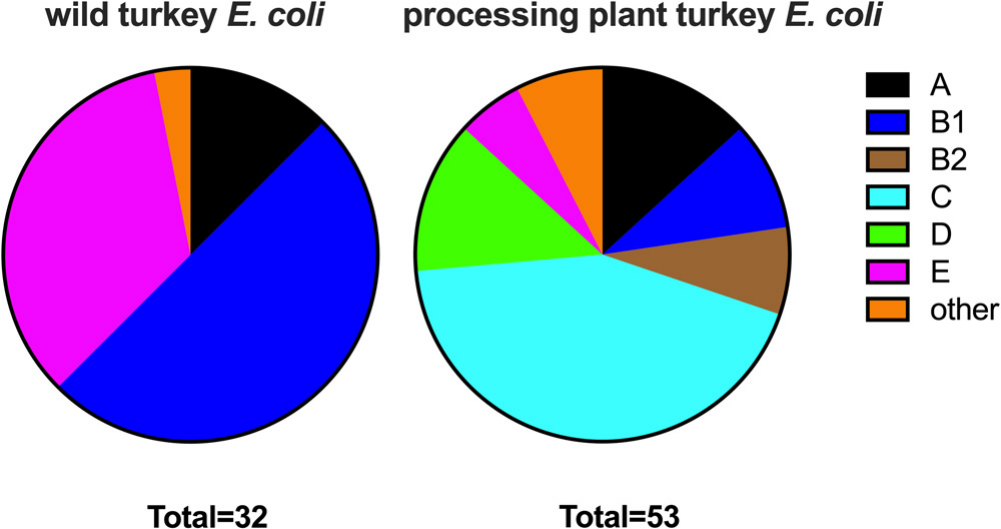
E. coli strains isolated from wild and factory-raised turkeys belong to distinct phylogroups. Strains were assigned to phylogroups according to Clermont PCR typing (96). Strains from factory-raised turkeys were more diverse and included those belonging to lineages traditionally thought to include pathogenic strains (lineages B2 and D).

As we were able to isolate E. coli colonies from the ceca of both wild and factory-raised turkeys, we were interested in further understanding the differences that may exist between these bacterial populations. We therefore performed phylogroup analysis to compare the diversities of E. coli lineages that were isolated from factory-raised and wild turkeys. Of E. coli strains isolated from wild turkeys, 29 of 30 strains belonged to group A, B1, or E, whereas none belonged to group B2, C, or D (Fig. 4 and Data Set S3). Strains isolated from factory-raised turkeys were more diverse, with all major phylogroups being represented. Several strains (9/50) belonged to cryptic clade I or II, which have been infrequently isolated in other studies. Seven strains were classified as group B2 or D, which are lineages that are commonly associated with extraintestinal pathogenic E. coli strains (27, 28). These results suggested that the pathogenic potential of the E. coli strains present in wild turkeys may be different from that of the strains present in factory-raised domestic turkeys.

A number of virulence factors have been identified in extraintestinal pathogenic E. coli. These include proteins essential for iron acquisition and for group 2 or group 3 capsule production (29–35). To determine if virulence-associated gene carriage differed between E. coli strains found in wild turkeys and those found in factory-raised turkeys, endpoint PCR was used to determine the carriage of three siderophore receptor genes (*iutA*, *iroN*, and *fyuA*) as well as the *kpsMT* genes involved in group 2 or group 3 capsule synthesis (Table 3). Nearly one-half (47%) of the E. coli strains isolated from wild turkeys carried the aerobactin receptor gene *iutA*. However, carriage of the salmochelin receptor gene *iroN*, the yersiniabactin receptor gene *fyuA*, or the capsule synthesis genes *kpsMT* was not observed in E. coli strains isolated from wild turkeys. Conversely, only 10% of strains isolated from the ceca of commercially produced turkeys contained *iutA*, whereas *iroN* was present in 22%, and *fyuA* was present in 4%. Capsule synthesis genes (*kpsMTII* or *kpsMTIII*) were present in 40% of strains isolated from factory-raised turkeys. The presence of virulence factors was not associated with any particular phylogroup, and several strains carried combinations of virulence factor genes (Table 3 and Data Set S3).

**TABLE 3:** Prevalence of virulence factor genes in E. coli strains isolated from the ceca of wild and factory-raised turkeys^*a*^

VF	No. of positive turkeys/total no. of turkeys	
	Wild	Factory raised
No VF	0/32	15/53
*iutA*	15/32	5/53
*iroN*	1/32	13/53
*fyuA*	0/32	7/53
*kpsII*	0/32	23/53
*kpsIII*	0/32	4/53
2 VFs	0/32	9/53
3 VFs	0/32	1/53
4 VFs	0/32	1/53

a VF, virulence factor.

## DISCUSSION

The essential role of the gut microbiota in maintaining animal and human health has been well established (36–39). Although diet is clearly an important selector for many functional guilds of microbes within the gut, host evolutionary history is thought to be a driving factor in determining the prevalence of specific microbial operational taxonomic units (OTUs) (40). Increasingly, evidence supports the theory that many animals coevolved with their microbial symbionts, giving both the host and microbe survival advantages (41–44).

In addition to diet, the intestinal microbiome of domestic farm animals (including poultry) is likely influenced by several factors, such as past and present exposure to antibiotics, exposure to the microbiome of the mother, and other microbes in their environment. The data presented here suggest that common production practices (potentially in combination with selective breeding) in modern poultry farming have resulted in a turkey microbiome in which beta diversity decreases from wild birds to free-ranging domestic birds to the highly monotaxic microbiota seen in commercially raised turkeys. The V4 sequencing results from this study are largely consistent with the results of a previous clone-based sequencing approach comparing the microbiota of wild turkeys and domestic turkeys (11).

The principal-component analysis (PCA) results presented in Fig. 1 clearly demonstrate that microbes from factory farm-raised, hobby farm-raised, and wild turkeys separate into distinct groups. In wild birds as well as hobby farm-raised birds, individual data points from different flocks were distributed throughout the groupings. However, our data from the factory farm-raised turkeys suggest that individual flocks may have perceptibly different microbiomes. Samples from factory farm flock 1 cluster separately from those of factory farm flock 2 according to the PCA (Fig. 1B). Distinct differences between factory flocks 1 and 2 are also evident in the taxonomic abundance data (Fig. 2) as well as Staphylococcus, Salmonella, and E. coli levels (Fig. 2 and 3).

As birds in factory flocks 1 and 2 were raised on standard commercial diets under similar housing conditions, we believe that the observed differences between these two flocks are most likely due to age differences between flock 1 (aged ∼6 weeks) and flock 2 (aged ∼18 weeks). Previous research has clearly demonstrated that the microbiome of developing turkeys undergoes significant changes over time (25, 45, 46). However, subtle differences in the microbiomes of commercial turkeys have also been observed on different farms (22, 45), which may be caused by variable local environmental conditions, including humans or other animals whose microbes may come into contact with the birds.

The composition of the gut microbiota in poultry likely influences a variety of beneficial characteristics, including immune system development and function (47–49). Domestic turkeys raised in commercial turkey production facilities are highly susceptible to a myriad of economically devastating bacterial, fungal, viral, and parasitic diseases (32). Previous research has shown that colonization by some commensal species of microbes prevents/inhibits colonization by pathogenic Campylobacter, Staphylococcus, and Salmonella (50–57) in poultry. We hypothesize that wild relatives of agriculturally important species may carry a heritable microbiome that inhibits colonization by common pathogens. Modern agricultural production practices have largely ignored the potential benefits of this natural microbiota, having instead relied on the widespread use of antibiotics to control pathogens.

While we have not yet established any specific mechanistic links between members of the normal flora and the abundance of specific pathogenic species, we observed that wild turkeys have higher levels of *Coriobacteriales* than hobby farm- or factory-raised domestic turkeys. Some *Coriobacteriales* produce hydroxysteroid dehydrogenase enzymes involved in the conversion of primary to secondary bile acids (58, 59). Bile salt conversion has demonstrated effects on the composition of the microbiome, colonization by intestinal pathogens, and immune responses in humans and livestock (58, 60–63). The growth of *Coriobacteriales* is stimulated by polyphenols found in diverse plants, and these bacteria metabolize them to phenolic compounds that have anti-inflammatory and immunomodulatory effects (59). *Coriobacteriales* are also especially prone to disruption by antibiotic treatment in mice (64). The diets of wild turkeys are free from agricultural antibiotics and likely contain diverse plant polyphenols. Whether members of this family are involved in colonization resistance to Campylobacter, Salmonella, or E. coli should be investigated further.

Suppression of avian-pathogenic E. coli in turkeys is an especially important priority for poultry producers. Therefore, it is notable that wild turkeys appeared to contain very little E. coli in their ceca. Furthermore, E. coli strains isolated from wild turkeys were dissimilar to those isolated from factory-raised birds, in terms of both phylogenetic lineage as well as the presence of specific virulence factors. Many of the E. coli strains isolated from wild turkeys contained the aerobactin receptor gene. Aerobactin is a proven virulence factor in extraintestinal avian infections (33); however, its role in these strains may be related to fitness in the highly competitive environment of the wild turkey intestinal tract. The absence of capsule synthesis genes and salmochelin and yersiniabactin production from strains isolated from wild turkeys may indicate that these strains are not prone to causing bloodstream infections or colonizing other organs. It is possible that bacteriocins, prophages, contact-dependent inhibition, or type 6 secretion systems of E. coli lineages established within the ceca of wild turkeys exclude invasion by avian-pathogenic strains frequently found in factory-raised poultry (65–68).

Due to common poultry production practices, microbes colonizing the intestinal tract of commercially raised poultry are minimally, if at all, influenced by the microbiome of the mother. The practice of hatching surface-sterilized eggs in incubators for multiple generations of birds has surely contributed to the loss of any heritable microbial taxa that may have coevolved with the wild turkey over millennia. Consequently, domestic poultry most likely obtain their microbiota almost exclusively from the environment found in the production facilities in which they are raised. Factors influencing the microbiome of these birds are limited to eggshells, litter, feed, and water (69–71). As poultry are often grown in the same production facilities generation after generation, microbial contamination from previous generations of birds is likely a significant factor influencing the microbiome of birds raised in high-density factory farms. The taxa and gene carriage of microbes that seed the microbiome of factory farm-raised birds have also been influenced by production practices such as feeding growth-enhancing antibiotics and feeds containing metals such as copper and zinc (72, 73). These have likely skewed the gut microbiota of poultry raised in large-scale production facilities toward taxa most capable of survival in modern turkey production facilities rather than microbiota contributing to the mutual survival of the host and microbe.

The transfer of microbiota from mother to infant has been best characterized in mammals. The transfer of maternal microbes to the mammalian young begins during the birthing process and continues through nursing and social interactions (74, 75). Coprophagy is common in many animal species, including turkeys (76–78), and it is common to see turkeys consuming cecal drops of their cage mates. This innate behavior in turkeys may have evolved to enable the bird-to-bird spread of beneficial microbiota within a flock. Recent work documents that the newly hatched young of some birds readily consume cecal drops, but not normal rectal feces, of their mothers. This consumption of maternal cecal drops by chicks was observed only during a short window of time (approximately the first month of life) (79). This behavior potentially facilitates the establishment of a beneficial, heritable gut microbiome from mother to chick.

The gut microbiome is perhaps one of the most complex biological communities. As in the analysis of any biological community, it is essential to consider the effects of dominant taxa as well as taxa that may have a relatively small but potentially important role in the community. The goal of this study was to identify potential changes/differences in the microbial composition of factory-raised turkeys compared to that of their wild predecessors. The results presented here demonstrate that the overall abundance of E. coli, Salmonella, Campylobacter, and Staphylococcus in wild turkeys is much lower than the levels commonly found in commercially raised turkeys. Furthermore, E. coli strains occupying the intestinal tract of wild turkeys appear distinct in both lineage and carriage of common virulence factors compared to strains commonly found in commercially raised turkeys. The strong correlation between bird provenance, increased microbial diversity, and low pathogen carriage warrants further research into the potential for mining the microbiome of free-ranging wild turkeys (as well as wild relatives of other agriculturally important species) in search of therapeutics or probiotics for use in controlling pathogens common in agricultural food production.

## MATERIALS AND METHODS

### Definition of turkey groups used in this study.

The term “wild turkey” can mean both a strain of turkey as well as the lack of domestication. In this study, we define wild turkey as a population of self-sustaining, wild, free-ranging birds. All wild turkeys sampled in this work were of the Rio Grande subspecies (Meleagris gallopavo *intermedia*) that have ranged freely for generations in the mountains of North Central Utah in the United States. Birds described as “free-range domestic turkeys” in this study are domesticated turkeys ranging freely outdoors. All free-range domestic turkeys in this study were from hobby farms where they were allowed to forage freely outdoors in both the summer and winter. The diet of all domestic free-range turkeys was supplemented with commercial poultry food by their owners. The term “factory-raised domestic turkey” refers to turkeys raised in commercial turkey production facilities. All turkeys in this group were of the Broad Breasted White variety. Although these factory-raised birds may fit the legal definition of “free-range” by virtue of their caging conditions, they were not considered free-ranging for the purposes of this study.

### Collection of cecal samples.

Some birds, including turkeys and chickens, produce two distinctly different types of feces. Cecal drops are a type of feces that the bird periodically excretes directly from the intestinal cecum (80). Previous work has demonstrated that the cecum contains the greatest microbial diversity found in the intestinal tract of poultry (81, 82). Additionally, the microbiota found in cecal drops is highly reflective of the microbiota found in cecal contents collected following sacrifice of the bird (83). The collection of cecal drops, which are easily distinguishable from normal feces, enables a simple, noninvasive method of obtaining a clear view of the cecal microbiota and eliminates the need to sacrifice (or even to come into contact with) study animals.

In this study, all samples were of cecal origin. Cecal drops from wild and free-ranging domestic turkeys were collected during the winter months following snowstorms. Sample collection immediately following snowstorms ensured that only fresh samples were collected and that the sample remained relatively uncontaminated by bacteria from the soil or other environmental sources. Cecal contents from one flock of factory-raised turkeys were collected from a turkey processing facility postmortem. Cecal drops from a second commercially raised flock were collected from the floor of the production facility. Sampling sites, bird age, and other details of sample origin are listed in Fig. S1 in the supplemental material.

### DNA preparation.

Following sample collection, all cecal contents were kept frozen until DNA isolation. DNA used for V4 sequencing was extracted from each sample using the Zymo Quick-DNA fecal/soil microbe 96 kit (catalog number D6011) according to the manufacturer’s instructions, including a bead homogenization step using a 2010 Geno/Grinder (Spex, Metuchen, NJ) at 1,750 rpm for 10 min. DNA was prepared for 16S rRNA gene V4 region sequencing based on an established protocol, with minor deviations (84). First, the V4 region of the 16S rRNA gene was amplified individually from each sample with the AccuPrime *Pfx* enzyme (Thermo Fisher Scientific, Waltham, MA, USA) in 20-μL volumes using a subset of the exact primer sequences described previously (84). PCR amplicons were normalized using the SequalPrep normalization kit (Applied Biosystems, Waltham, MA, USA) and pooled into groups of 96 reaction mixtures, and fragments in the range of 250 to 450 bp were purified using a BluePippin (Sage Science, Beverly, MA) selection step. Equimolar normalization of each pool and sequencing were performed at the BYU DNA Sequencing center on a partial 2-by-250 lane (v2) of a HiSeq 2500 platform (Illumina, Inc., San Diego, CA). Laser complexity was ensured by including at least 10% of each lane with shotgun sequencing libraries for other bacterial genomes.

### Sequence analysis.

Sample reads were demultiplexed on the Illumina platform and analyzed using QIIME2 (85, 86) and R. Briefly, reads were trimmed to maximize the quality scores of each nucleotide position. DADA2 (87) was used to denoise, dereplicate, and call amplicon sequence variants (ASVs), and taxonomy was assigned to the ASVs using GreenGenes classifier 13_8_99 (88). ASV tables were filtered to 13,000 reads per sample, and differences between groups were determined by PERMANOVA (89) of weighted and unweighted UniFrac distances (90, 91). To permit calculating UniFrac distances, we built a phylogenetic tree with fasttree2 (92) based on mafft alignment (93). Differences in OTU abundances between samples were determined using ANCOM (94). Abundances of individual OTUs were manually analyzed based on the taxonomic assignments, which were assigned to OTUs using QIIME2 q2-feature-classifier (95). Alpha diversity metrics were defined using QIIME2, and differences in alpha diversity metrics between sampling locations were determined by a Kruskal-Wallis test.

### Determination of relative E. coli DNA levels in cecal samples.

A qPCR-based assay was designed to estimate the relative abundance of E. coli DNA in each cecal sample based on the detection of the *ybbW* gene, which is found exclusively in E. coli (26). For qPCR experiments, total DNA was isolated from cecal samples using the Qiagen blood and tissue DNA kit as directed by the manufacturer. DNA samples were diluted to a concentration of 100 ng/μL. IDT PrimeTime gene expression master mix was used in all qPCR assays. Thermocycling was performed as suggested by the manufacturer (40 cycles of 95°C of denaturation for 15 s followed by 57°C of annealing/amplification for 1 min) using an ABI StepOnePlus real-time PCR system. The qPCR primers and probe were designed using IDT PrimerQuest and manufactured by Integrated DNA Technologies. Primer and probe sets used in this study are listed in Table 4. The efficiency and reproducibility of amplification were verified by generating a standard curve using doubling dilutions of positive-control DNA. Negative controls consisted of reaction mixtures with DNA elution buffer rather than DNA. Each sample was tested in duplicate. Purified DNA from pooled E. coli strains was used as a positive control.

**TABLE 4:** Primers used in this study

Primer or probe	Sequence	Target, expected size (bp)
invA 1F	GTGAAATTATCGCCACGTTCGGGCAA	*invA*, 284
invA 1R	TCATCGCACCGTCAAAGGAACC	

iutA F	CTGCAGTACTCCGATCGGCTG	*iutA*, 470
iutA R	TGGTTGGAGGTAAAGCGCTCATG	

iroN R	TGTCGGTACAGGCGGTTCGTC	*iroN*, 814
iroN F	CTCTGGTGGTGGAAGCCACC	

fyuA F	ACGGCTTTATCCTCTGGCCTTGG	*fyuA*, 877
fyuA R	TGAAAACCCAGTCATCGGTGG	

kpsII F	GCGCATTTGCTGATACTGTTG	*kpsMTII*, 581
kpsII R	AGGTAGTTCAGACTCACACCT	

kpsIII F	TCCTCTTGCTACTATTCCCCCT	*kpsMIII*, 390
kpsIII R	AAGGCGTATCCATCCCTCCTAAC	

chuA.1b	ATGGTACCGGACGAACCAAC	*chuA*, 288
chuA.2	TGCCGCCAGTACCAAAGACA	

AceK.f	AACGCTATTCGCCAGCTTGC	*arpA*, 400
ArpA1.r	TCTCCCCATACCGTACGCTA	

yjaA.1b	CAAACGTGAAGTGTCAGGAG	*yjaA*, 211
yjaA.2b	AATGCGTTCCTCAACCTGTG	

TspE4C2.1b	CACTATTCGTAAGGTCATCC	*TspE4.C2*, 152
TspE4C2.2b	AGTTTATCGCTGCGGGTCGC	

ArpAgpE.f	GATTCCATCTTGTCAAAATATGCC	Group E *arpA*, 301
ArpAgpE.r	GAAAAGAAAAAGAATTCCCAAGAG	

trpAgpC.1	AGTTTTATGCCCAGTGCGAG	Group C *trpA*, 219
trpAgpC.2	TCTGCGCCGGTCACGCCC	

ybbW 1F	TGATTGGCAAATCTGGCCG	qPCR of *ybbW*
ybbW 1R	CGTTGACCAGCCAGAAGATTAAG	

ybbW Probe	56-FAM/AAGCCCGGT/ZEN/AGAGAAAGGCCTAAC/3IABkFQ^*a*^	Probe for qPCR of *ybbW*

a FAM, carboxyfluorescein; 3IABkFQ, 3’ Iowa Black FQ.

### Isolation and genotyping of E. coli strains.

E. coli strains present in cecal samples were isolated by homogenizing a portion of the sample in sterile phosphate-buffered saline (PBS) and plating the sample onto MacConkey agar, followed by growth at 37°C for 24 h. Colonies with characteristic E. coli morphology were then restreaked and verified as E. coli by PCR targeting the *ybbW* gene. Total DNA was isolated from individual colonies using a minigenomic DNA kit for blood and cultured cells (IBI Scientific). Putative E. coli strains were assigned to phylogroups using the Clermont quadruplex assay, with additional PCR tests to distinguish group C or group E when warranted, as previously described (96).

The presence or absence of genes associated with the virulence of avian-pathogenic E. coli (*iutA*, *iss*, *iroN*, *fyuA*, *kpsMTII*, and *kpsMTIII*) was determined by PCR. These reactions were performed using approximately 100 ng genomic DNA as the template and 20 pmol of each primer in OneTaq master mix. The conditions were 94°C for 3 min followed by 30 cycles of 94°C for 15 s, 57°C for 15 s, and 68°C for 45 s and a final extension step at 68°C for 5 min. The *fyuA*, *kpsII*, and *kpsIII* reactions were multiplexed, while the *iutA*, *iroN*, and *iutA* reactions were run individually. Primers used for these reactions are listed in Table 4.

### Detection of Salmonella DNA in cecal samples.

The presence or absence of Salmonella sp. in cecal sample DNA was determined using a semiquantitative PCR assay using primers targeting the *invA* gene (Table 4), which has previously been demonstrated to specifically detect most Salmonella strains (97). The conditions used for these PCR tests were identical to the ones for the E. coli virulence genotyping described above, except that the annealing temperature was 56°C.

### Data availability.

Sequences were deposited to the National Center for Biotechnology Information Sequence Read Archive under BioProject accession number PRJNA786944.
